# Multidrug Resistance Protein-4 Influences Aspirin Toxicity in Human Cell Line

**DOI:** 10.1155/2015/607957

**Published:** 2015-09-27

**Authors:** Isabella Massimi, Ambra Ciuffetta, Flavia Temperilli, Francesca Ferrandino, Alessandra Zicari, Fabio M. Pulcinelli, Maria Pia Felli

**Affiliations:** ^1^Department of Experimental Medicine, Faculty of Medicine and Surgery, Sapienza University of Rome, 00161 Rome, Italy; ^2^Department of Molecular Medicine, Faculty of Medicine and Surgery, Sapienza University of Rome, 00161 Rome, Italy

## Abstract

Overexpression of efflux transporters, in human cells, is a mechanism of resistance to drug and also to chemotherapy. We found that multidrug resistance protein-4 (MRP4) overexpression has a role in reducing aspirin action in patients after bypass surgery and, very recently, we found that aspirin enhances platelet MRP4 levels through peroxisome proliferator activated receptor-*α* (PPAR*α*). In the present paper, we verified whether exposure of human embryonic kidney-293 cells (Hek-293) to aspirin modifies MRP4 gene expression and its correlation with drug elimination and cell toxicity. We first investigated the effect of high-dose aspirin in Hek-293 and we showed that aspirin is able to increase cell toxicity dose-dependently. Furthermore, aspirin effects, induced at low dose, already enhance MRP4 gene expression. Based on these findings, we compared cell viability in Hek-293, after high-dose aspirin treatment, in MRP4 overexpressing cells, either after aspirin pretreatment or in MRP4 transfected cells; in both cases, a decrease of selective aspirin cell growth inhibition was observed, in comparison with the control cultures. Altogether, these data suggest that exposing cells to low nontoxic aspirin dosages can induce gene expression alterations that may lead to the efflux transporter protein overexpression, thus increasing cellular detoxification of aspirin.

## 1. Introduction

Exposure of eukaryotic cells to drugs can trigger modifications in the expression of mechanisms susceptible to favour their elimination. In hepatocytes, this is most often related to the transient induction of the transcriptional regulation of genes by nuclear receptors [1–4].

Members of the ATP-binding cassette (ABC) transporter superfamily are widely recognized as major contributors to controlling drug distribution and pharmacokinetics and the acquisition of anticancer drug resistance [5].

In cancer cells, efflux transporters' overexpression is one mechanism of resistance to chemotherapy [6, 7].* In vitro* exposure of cells for a prolonged period of time to anticancer agents shows a correlation between increasing concentrations of drugs and overexpression of transporters [3, 8].

This approach can be applied to many other drugs to identify the transporter responsible for their efflux and responsible for resistance mechanism.

A member of the MRP/ABCC subfamily of ATP-binding cassette transporters, which are capable of pumping a wide variety of endogenous (including cyclic nucleotides) and xenobiotic organic anionic compounds out of the cell, is MRP4, which can be upregulated to reduce intracellular organic anion toxicity or cholestasis [9].

We previously demonstrated that aspirin is a substrate for MRP4 in human platelets [10], and it was confirmed that both aspirin and its salicylic acid (ASA) are substrates of mouse ABCC4 (MRP4) [11].

One of our recent studies showed aspirin ability to influence megakaryocytic gene expression, leading to upregulation of MRP4 in human platelets through the activation of the nuclear receptor PPAR*α* [12], suggesting that even aspirin can activate mechanisms that favour its elimination and consequently reduce its toxic effect.

Acute salicylate poisoning is a common medical emergency, which carries a high mortality [13–15]. Salicylate poisoning remains a clinically hazardous therapeutically acquired intoxication at any age [16].

Aspirin poisoning is clearly dose related to increase toxicity in human subjects [17]. Daily aspirin use, whether regular strength or low dose, results in reduction in cancer incidence and mortality, although potential side effect profiles must be considered. It was suggested that one of the mechanisms by which aspirin is chemopreventive for cancer is its capability of inhibiting tumor cell proliferation [18]. In fact, aspirin toxicity results from the perturbation of the cell cycle and ultimately causes necrosis [19].

In this paper we showed that aspirin-dependent MRP4 overexpression effectively reduces the cytosolic concentration of aspirin in cells exposed to increasing concentrations of this drug, providing a simple resistance mechanism.

We have now examined how human cells would respond to stepwise exposure to increasing concentrations of drug either in basal or in enhanced efflux protein transporter expression (in the absence or in the presence of a detectable efflux transporter). Indeed, we observed reduced aspirin toxicity when the expression of MRP4 transporter is higher.

## 2. Material and Methods

### 2.1. Cell Line and Culture Conditions

Human embryonic kidney-293 cells, Hek-293 cell line, were maintained in DMEM supplemented with 10% heat-inactivated fetal bovine serum, 20 mM L-glutamine, 100 units/mL of penicillin G sodium, and 100 *μ*g/mL streptomycin sulfate in a humidified atmosphere containing 5% CO_2_ at 37°C. Cells were treated with aspirin, either to induce MRP4 expression (50 *μ*M for 48 h) or to induce cell death (5 mM for 24 h) (SIGMA Chemicals Company, St. Louis, MO) to study either gene expression changes or aspirin toxicity, respectively. Control cells were treated with vehicle.

### 2.2. DNA Constructs and Transfection

The PCDNA 3.1-MRP4 vector expressing the human MRP4 protein was from Dr. Rius et al. [20]. Cell transfection was performed as previously described [21]; briefly, Hek-293 (1 × 10^6^ cells plated in 60 mm dishes) cells were transfected with the indicated amount (0.5 *μ*g) of MRP4 expression vectors by TransFectin Lipid Reagent (Bio-Rad Laboratories, Italy). An equivalent amount of transfection reagent (TransFectin Lipid Reagent, Bio-Rad), with PCDNA 3.1, was added as mock control. 24 hours after transfection, cells were treated with a high concentration of aspirin.

### 2.3. Cell Death Assay

In order to evaluate aspirin-dependent toxicity, trypan blue assay and 7-AAD staining were performed.

At the end of the treatment, cells were detached by trypsinization and incubated with trypan blue and counted for stained and unstained cells.

For FACS analysis, under different experiment conditions, 1 × 10^6^ cultured Hek-293 cells were freshly harvested and incubated for 10 minutes in the dark with 5 *μ*L/test of 7-AAD (BD Pharmingen) [21]. Within 1 h after dye incubation, the cells were analysed on FACSscan (Becton Dickinson, Mountain View, CA) using Cell Quest software (Becton Dickinson).

### 2.4. High-Performance Liquid Chromatography Analysis

Quantitative analysis of plasma concentrations of aspirin was performed using HPLC according to Cerletti et al., 2003 [22]. Cells were washed twice with cold PBS, collected, and centrifuged at 400 g for 10 min. After extraction with hexane (SIGMA Chemicals Company, St. Louis, MO), aspirin was quantified by high-performance liquid chromatography (HPLC) with ultraviolet detection at 229 nm. Aspirin concentration and peak-height ratios were linearly related up to 20 *μ*g/mL. The lower limit of sensitivity was 0.1 *μ*g/mL. The average recovery of aspirin was 27%.

### 2.5. Protein Extraction and Western Blot

In order to analyze MRP4 protein, cells were washed twice with cold PBS, collected, and centrifuged at 400 g for 10 min. Cell pellets were then resuspended in lysis buffer (RIPA buffer: 10 mM Tris-HCl (pH 7.6), 160 mM NaCl, 1 mM EGTA, 1% deoxycholic acid, 1% Triton, and 0.1% SDS) with protease inhibitors, incubated on ice for 30 min, and centrifuged at 12,000 g for 30 min; the supernatant was then collected.

Protein extracts (30 *μ*g) were incubated at 37°C for 30 min [20] and separated on 4–12% SDS-PAGE gel, blotted onto PVDF membrane (GE Healthcare, Milano, Italy), and probed with rat anti-MRP4 (Alexis, Plymouth Meeting, Pennsylvania) and mouse anti-actin (Santa Cruz Biotechnology, Santa Cruz, CA) monoclonal antibodies. Immunoreactive bands were visualized by enhanced chemoluminescence (PerkinElmer, Waltham, MA, USA).

### 2.6. RNA Preparation and Real-Time PCR Analysis

Total RNA from human cell lines was extracted using TRIzol reagent (Invitrogen, San Diego, CA).

For mRNA detection 1 *μ*g of total RNA was transcribed using the GeneAmp Gold RNA PCR Reagent Kit pAW109 (Applied Biosystems, Warrington, UK) according to the manufacturer's suggestion.

The analysis of gene expression was carried out with Q-RT-PCR using TaqMan Master Mix and TaqMan Assay Reagents (Applied Biosystems, Warrington, UK).

The amplification, monitored using ABI Prism 7900 Sequencer Detector (Applied Biosystems, Foster City, CA), was as follows: 50°C for 2 min, 95°C for 10 min, 95°C for 15 sec, and 60°C for 1 min; the latter two temperatures were repeated for 40 cycles.

All amplification reactions were performed in duplicate using 25 ng of cDNA.

Changes in MRP4 and *β*-actin mRNA amounts were quantified by using ΔΔCt method for relative quantization of gene expression using SDS software version 2.3 (Applied Biosystems, Warrington, UK).

### 2.7. Statistical Analysis

Data are presented as mean ± SD. The level of significance was determined by paired, 2-tailed Student's *t*-test (KaleidaGraph software 3.6). Results were considered statistically significant if a *P* value of less than 0.05 was reached.

## 3. Results

### 3.1. Influence of Aspirin on Cell Viability

**Figure 1:**
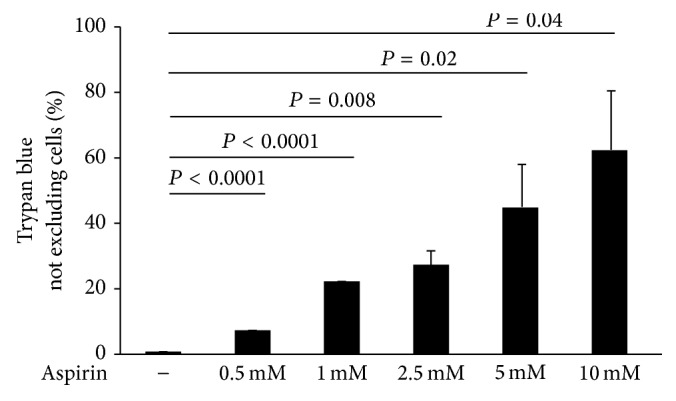
Cells survival depends on the dose of aspirin. Cell survival of either untreated or aspirin treated (from 0.5 mM to 10 mM) Hek-293 cells for 24 hours. Trypan blue exclusion test analysis was used to analyze cell death (“trypan blue-not excluding”). “Trypan blue-not excluding” cell analysis is reported as percentage of dead cells, in aspirin treated compared to untreated cells. Data are reported as mean ± SD of 3 experiments performed.

As a preliminary step in this work, we examined to what extent cells gone through the process of selection by aspirin showed a distinct pattern of viability compared to untreated cells (control). Human embryonic kidney-293 cells (Hek-293) were incubated with high concentrations of aspirin (from 0.5 mM to 10 mM, for 24 h) and then counted and subjected to trypan blue assays (Figure 1). A dose-response analysis through cell numeration showed that aspirin markedly reduced cell viability, suggesting that high concentrations (0.5–10 mM) of aspirin have a toxic effect. This was demonstrated by trypan blue assays; in fact, aspirin causes cell death in a dose-dependent manner: 24 h treatment with high concentrations of this drug was cytotoxic for a large proportion of Hek-293 cells, while low concentrations were less effective (Figure 1).

### 3.2. MRP4 Expression in Aspirin Treated Hek-293 Cells

We recently demonstrated that aspirin is a target for MRP4 and, in* in vitro* treatment, it increases MRP4 expression [10, 12]. These studies suggest that MRP4 might be important for aspirin outward transport by cells and for aspirin cell detoxification.

To confirm whether aspirin modulates MRP4 expression in Hek-293 cells, we analyzed mRNA and protein expression both in control and in aspirin treated cells.

**Figure 2:**
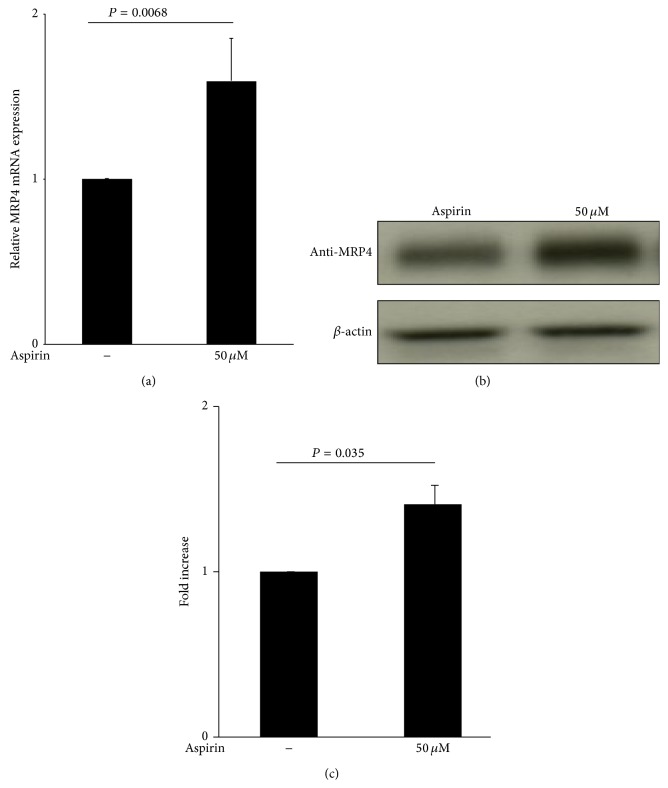
Aspirin stimulates endogenous MRP4 mRNA and protein expression in Hek-293 cells. (a) Q-RT-PCR analysis of endogenous MRP4 *α* expression after 48 h of treatment with 50 *μ*M aspirin. Data were normalized with *β*-actin expression and reported as mean ± SD of increasing fold, compared to control (*n* = 3). (b) Representative western blot, of 3 experiments performed, of endogenous MRP4 expression after 48 h treatment with 50 *μ*M aspirin. Aspirin untreated cells were used as control cells. (c) Densitometric analysis of western blot is reported as mean ± SD of increasing fold, compared to control (*n* = 3).

Hek-293 cells treated with low-dose aspirin (50 *μ*M for 48 h) showed higher MRP4 mRNA levels compared to mock culture (Figure 2(a)) suggesting a positive transcriptional control.

In the same experimental conditions, western blot analysis revealed an increase of MRP4 protein expression in cells treated with aspirin (50 *μ*M for 48 h) (Figure 2(b)).

Densitometry analysis showed a statistically significant higher induction of MRP4 protein expression, which is enhanced more than 30% in comparison with untreated cells (Figure 2(c)).

Aspirin-dependent MRP4 overexpression may be important to reduce drug cytosolic concentration, leading to reduced aspirin toxicity.

### 3.3. Influence of High Concentrations of Aspirin on Control and Aspirin-Pretreated Cells

Recent studies showed that eukaryotic cells drug exposure modulates expression of mechanisms favouring their elimination.

To study whether this mechanism is evident also after aspirin treatment, Hek-293 cells were preincubated with aspirin (50 *μ*M) and after 48 h they were treated with high concentrations of aspirin (5 mM for 24 h). After these incubations, both control and pretreated cells were counted and subjected to trypan blue dye exclusion and flow cytometry (FACS) analysis with 7-AAD.

**Figure 3:**
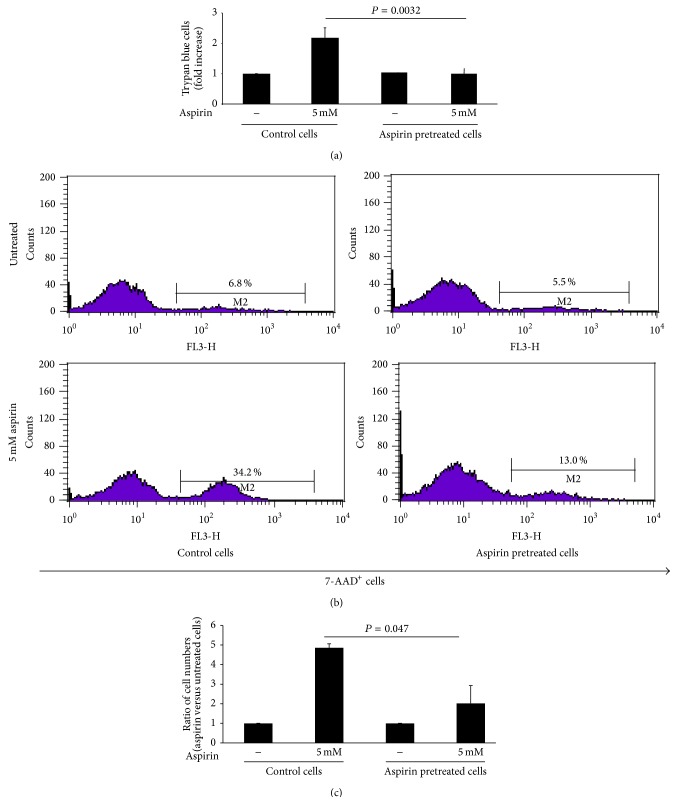
Aspirin pretreatment is important for reduction of its toxicity. Cell death in Hek-293 cells, pretreated with 50 *μ*M of aspirin (aspirin pretreated cells) or with vehicle (control cells) for 48 h. In both conditions, toxicity was evaluated in untreated and aspirin treated (5 mM) cells 24 h later. (a) Expression of “trypan blue-not excluding” cells is evaluated as percentage of dead cells in aspirin (5 mM) treated cells compared with control cells. Data were reported as mean ± SD of 3 experiments (NS: not significant). (b) Dead cell profile, representative flow cytometry histograms of Hek-293 cells (1 × 10^6^) stained with 7-AAD, out of three experiments performed. Left and right panels represent control and aspirin pretreated cells, respectively. Percentages of 7-AAD^+^ cells are indicated in each panel. (c) Ratio of the total numbers of 7-AAD^+^ cells in 5 mM aspirin treated versus matched untreated cells, both in control and in aspirin pretreated cells. Data were reported as mean ± SD of 3 experiments performed; *P* = 0.046 indicates significant differences in aspirin samples of control versus pretreated cells.

Trypan blue assay showed that, in cells directly treated with 5 mM aspirin for 24 h, cell viability was markedly reduced in control cells, while it was almost unchanged in comparison with 48 h aspirin pretreated cells (Figure 3(a)).

Interestingly, since staining with 7-AAD is more sensitive to cellular damage, and to evaluate the effect, we performed FACS analysis using 7-AAD, measuring the percentage of dead cells with compromised membranes gating on the whole cell population (Figure 3(b)). As shown in the upper panels, no differences in 7-AAD staining, before treatment, were observed between control (6.8%) and pretreated cells (5.5%). Instead, the lower panels of Figure 3(b) show an increase of dead cells in response to high-dose aspirin (7-AAD^+^ cells) in control cells (5-fold increase). On the other hand, in aspirin pretreated cells (50 *μ*M for 48 h) still overexpressing MRP4 (as shown in Figure 2(b)), a lower percentage of dead cells (control 34.2% versus pretreated 13.0%) was observed in response to high-dose aspirin. Moreover, we analyzed the absolute number of 7-AAD positive over total cells, in both control and pretreated cell samples. The results show that the ratio of total numbers of 7-AAD positive cells in aspirin (5 mM) treated samples versus matched untreated cells was reduced in a statistically significant way, when cells were pretreated with low doses of aspirin (50 *μ*M for 48 h) (Figure 3(c)).

The data could suggest that aspirin is able to induce gene expression changes, enhancing MRP4 expression in aspirin pretreated cells, which might favour aspirin transport outside the cells, leading to reduction of drug induced cell toxicity.

### 3.4. MRP4 Protects Hek-293 from Aspirin-Dependent Cytotoxicity

**Figure 4:**
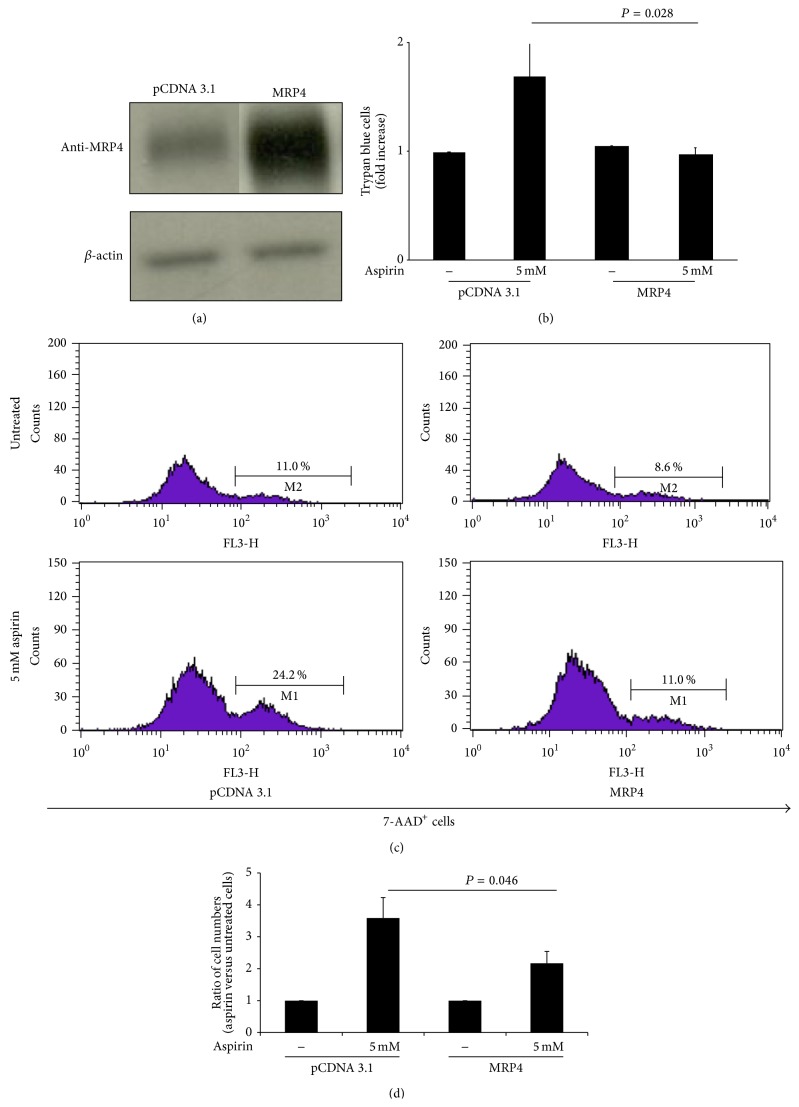
MRP4 expression is relevant to reduce aspirin toxicity. Cell death in Hek-293 transfected cells (MRP4 cells). pCDNA3.1 transfected cells were used as control (pCDNA3.1). Aspirin toxicity was evaluated after 24 h aspirin (5 mM) treatment, with trypan blue exclusion test. (a) Multidrug resistance protein-4 (anti-MRP4) expression in Hek-293 cell lines is shown in the first lane (PCDNA 3.1); MRP4 transfected cultures are shown in the second lane (MRP4). A representative experiment, of 3 performed, is presented. (b) Expression of “trypan blue-not excluding” cells is evaluated as percentage of cell death in aspirin treated cells (5 mM) compared with control cells. Data were reported as mean ± SD of 3 experiments performed (NS: not significant). (c) Dead cell profiles, representative flow cytometry histograms of Hek-293 cells stained with 7-AAD, out of three performed experiments. The panel represents pCDNA3.1 and MRP4 transfected cells. Numbers in histograms indicate percentage of 7-AAD^+^ cells. (d) Ratio of the total numbers of 7-AAD^+^ cells in 5 mM aspirin treated versus matched untreated cells, both in pCDNA3.1 and in MRP4 transfected cells. Data were reported as mean ± SD of 3 experiments performed; *P* = 0.046 indicates significant differences in aspirin samples of pCDNA3.1 versus MRP4.

To confirm that MRP4 upregulation is responsible for the reduced aspirin toxicity, we explored the effects of exogenous MRP4 overexpression in Hek-293 cells. These cells were transfected either with pCDNA 3.1 (pCDNA 3.1), used as a control, or with MRP4 expression vector (MRP4) and, further, incubated for 24 h with high concentration of aspirin (5 mM). Afterwards, a comparative study of transfected cells, with trypan blue dye assay and FACS analysis using 7-AAD staining, was performed. The efficiency of the transfection was monitored by western blot analysis confirming MRP4 protein overexpression in transfected cells (Figure 4(a)).

Trypan blue assay showed that cell viability was markedly reduced in control cells when exposed to 5 mM aspirin for 24 h, while it remains unchanged in MRP4 transfected cells subjected to the same treatment (Figure 4(b)).

A comparative analysis by FACS of pCDNA 3.1 versus MRP4 transfected cells was performed (Figure 4(c)). In aspirin treated cells (5 mM), the percentage of 7-AAD stained cells was increased in pCDNA 3.1 transfected cells, rising from 11.0% to 24.2%, while in MRP4 overexpressing cells the percentage of 7-AAD stained cells (7-AAD^+^ cells) was slightly enhanced (from 8.6% to 11.0%).

Under aspirin treatment (5 mM), the ratio of total numbers of 7-AAD positive cells in MRP4 versus matched pCDNA3.1 transfected cells was decreased, as observed in three experiments performed (Figure 4(d)).

Altogether, these data support the hypothesis that MRP4 overexpression is important to reduce aspirin-selective cell death. The protective effect may depend on the MRP4-induced aspirin extrusion from the cell.

### 3.5. Accumulation of Aspirin in Control and MRP4 Transfected Cells

To analyze whether MRP4 is able to modulate aspirin transport outside the cell, we compared intracellular accumulation of aspirin in MRP4 versus pCDNA 3.1 transfected cells both treated with 5 mM aspirin for 24 h.

**Figure 5:**
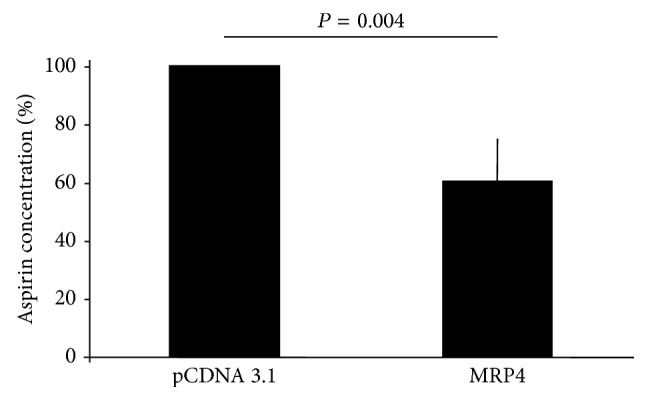
A reduced aspirin cytoplasmatic concentration in MRP4 transfected Hek-293 cells. Percentage of aspirin cytoplasmatic concentration in MRP4 transfected cells (MRP4) after aspirin treatment (5 mM for 24 h) in comparison with those found in control cells (pCDNA 3.1). The results are expressed as mean ± SD of 3 experiments performed.

After such treatment, we analyzed cytosolic aspirin accumulation using a selective and sensitive method. As shown in Figure 5, aspirin accumulation is reduced, about 2 times less, in MRP4 transfected compared to pCDNA 3.1 cells (Figure 5). These data demonstrate an inverse correlation between transporter and intracellular substrate level, since very high MRP4 expression is directly related to a reduced intracellular aspirin concentration.

## 4. Discussion

Many reports outlined a close correlation between increased MRP transporters expression and impairment of pharmacological treatment.

In the present study, we have shown that exposure of eukaryotic cells to aspirin, an MRP4 target [10, 11], can deeply modify the expression of this efflux transport protein, in a human cell line, Hek-293. Our results suggest that such modifications can result in enhanced aspirin transport outside the cells, leading to a reduction of the toxic effect of this drug.

Such relationship between drug toxicity and MRP4 upregulation is already reported in mouse. In fact, after* in vivo* perfluorooctanoic acid and perfluorodecanoic acid administration, liver mounts a compensatory hepatoprotective response, leading to a marked increase of MRP4 expression [23], in order to reduce drug toxicity.

As aspirin toxicity results in the perturbation of the cell cycle and ultimately causes necrosis [19], we investigated, first, through a dose-response curve, the effect of high-dose aspirin treatment, and we showed that aspirin is able to increase cell death in Hek-293 cell line, at similar concentrations to those found by others [19].

In our recent study, we showed that also aspirin is able to modulate MRP4 expression in human platelets, and we speculated that the limited drug capacity in reducing platelet function, observed in aspirin long-term treated patients [24], could be due to a drug-dependent MRP4 upregulation [12]. Aspirin influences megakaryocytic gene expression leading to upregulation of MRP4 in human platelets, suggesting that even aspirin can activate mechanisms that favour its elimination, thus consequently reducing its toxic effect [12].

In agreement with this previous report, aspirin-dependent MRP4 upregulation was confirmed also in Hek-293 cells, widening the spectrum of the cells subjected to this mechanism. In fact, aspirin treatment increases MRP4 mRNA and protein expression levels for 48 hours in Hek-293 cells. This upregulation induced also by low nontoxic aspirin dosage can be important to regulate aspirin effect. Indeed, pretreatment with low doses of aspirin reduces its toxicity by decreasing the percentage and absolute numbers of nonviable cells, suggesting the induction of a protective mechanism. We cannot exclude the contribution of a mechanism of cell selection operated by aspirin. This may affect drug intended and drug side effects in many tissue districts since MRP4 is ubiquitously expressed, including hematopoietic cells [25]. Furthermore, modulation of aspirin effects may be important in long-term daily aspirin use.

Several studies show that acute salicylate poisoning is a common medical emergency which leads to a high mortality [13–15] and aspirin poisoning is clearly dose related in order to increase toxicity in human subjects [17].

The direct connection between aspirin-induced MRP4 expression and cell toxicity was further addressed by overexpressing MRP4 extruding protein in our cell line. Indeed, MRP4 high levels are well correlated with reduced numbers of 7-AAD positive cells. In this study we demonstrate that aspirin-dependent MRP4 upregulation is important to reduce intracellular aspirin concentration, by enhancing its transport. In fact, MRP4 overexpression leads to cells drug detoxification in a more efficient manner in human Hek-293.

To confirm that MRP4 is the aspirin transporter involved in such phenomenon, we compared cellular accumulation of aspirin in MRP4 transfected cells versus control cells treated with aspirin (5 mM for 24 h). Indeed, aspirin showed a reduced cytosolic accumulation in MRP4 transfected compared to control cells.

Our data support the evidence that aspirin-dependent overexpression of MRP4 is a cell adaptation to what seems to be a major toxic stress [26].

With our results, we can also suggest that, in the future, it should be important to study a possible correlation between the treatment with aspirin and that with chemotherapeutic agents, in order to verify drug efficiency.

## 5. Conclusion

In conclusion, this study demonstrates that aspirin, at low nontoxic drug dosage, is able to activate a positive transcriptional control of the MRP4/ABCC4 transporter gene, in human cells, thus enhancing the activity of the mechanism susceptible to increase aspirin efflux.

We speculate that our data are suggestive of the ability of low-dose aspirin to confer clinical drug resistance, particularly in long-term treated patients.
